# A Triterpenoid Lupeol as an Antioxidant and Anti-Neuroinflammatory Agent: Impacts on Oxidative Stress in Alzheimer’s Disease

**DOI:** 10.3390/nu15133059

**Published:** 2023-07-07

**Authors:** Jun Sung Park, Inayat Ur Rehman, Kyonghwan Choe, Riaz Ahmad, Hyeon Jin Lee, Myeong Ok Kim

**Affiliations:** 1 Division of Life Sciences and Applied Life Science (BK21 Four), College of Natural Science, Gyeongsang National University, Jinju 52828, Republic of Korea; jsp@gnu.ac.kr (J.S.P.); inayaturrehman201516@gnu.ac.kr (I.U.R.); k.choe@gnu.ac.kr (K.C.); riazk0499@gnu.ac.kr (R.A.); lhj4912@gnu.ac.kr (H.J.L.); 2 Department of Psychiatry and Neuropsychology, School for Mental Health and Neuroscience (MHeNs), Maastricht University, 6229 ER Maastricht, The Netherlands; 3 Alz-Dementia Korea Co., Jinju 52828, Republic of Korea

**Keywords:** Alzheimer’s disease (AD), reactive oxygen species (ROS), oxidative stress (OS), neuroinflammation, Lupeol, antioxidant

## Abstract

Alzheimer’s disease (AD) is the most common neurodegenerative disease illustrated by neuronal dysfunctions, leading to memory weaknesses and personality changes mostly in the aged population worldwide. The exact cause of AD is unclear, but numerous studies have addressed the involvement of oxidative stress (OS), induced by reactive oxygen species (ROS), to be one of the leading causes in developing AD. OS dysregulates the cellular homeostasis, causing abnormal protein and lipid metabolism. Nutrition plays a pivotal role in modulating the antioxidant system and decreases the neuronal ROS level, thus playing an important therapeutic role in neurodegenerative diseases, especially in AD. Hence, medicinal herbs and their extracts have received global attention as a commercial source of antioxidants Lupeol. Lupeol is a pentacyclic triterpenoid and has many biological functions. It is available in fruits, vegetables, and medicinal plants. It has shown effective antioxidant and anti-inflammatory properties, and higher blood–brain barrier permeability. Also, the binding and inhibitory potentials of Lupeol have been investigated and proved to be effective against certain receptor proteins and enzymes in AD studies by computational molecular docking approaches. Therefore, AD-related research has gained interest in investigating the therapeutic effects of Lupeol. However, despite its beneficial effects in AD, there is still a lack of research in Lupeol. Hence, we compiled in this analysis all preclinical research that looked at Lupeol as an antioxidant and anti-inflammatory agent for AD.

## 1. Introduction

Alzheimer’s disease (AD) is an irreversible [1] neural malfunction, leading to changes in personality, cognition, and memory in older people over time [2]. Additionally, globally, AD causes a significant impact not only to the patient and their caretakers, but also to society and the economy [3]. The main pathology of AD is the accumulation of amyloid-beta (Aβ) peptides and hyperphosphorylated tau proteins [4]. Furthermore, other factors such as abnormalities in cholinergic neurons, inflammatory cascades, and oxidative stress (OS) are contributing factors to the initiation and propagation of AD pathology [5]. OS is known to disturb the cellular metabolic system of biomolecules such as proteins, lipids, and nucleic acids, creating a load of cellular dysfunction [6]. However, there is no cure for AD and the available treatments resulted in limited effectiveness [7]. Therefore, the preventive potential of nutrition is one of the major and leading possible solutions in ameliorating AD pathology [8]. For example, a balanced diet containing antioxidants has been reported to exhibit neuroprotective properties, by eliminating the reactive oxygen species (ROS), in AD [9,10]. Triterpenoids are reported to have promising health effects over the past decades and play a versatile role in different types of chronic disorders [11], including neuronal dysfunction such as AD [12]. Particularly, Lupeol, a pentacyclic triterpenoid, has numerous biochemical properties and it is available in a wide range of vegetables and fruits such as cucumber, white cabbage, strawberry, and mango [13,14], through the mevalonate pathway [11]. Also, it has been shown to have antioxidant and neuroprotective properties in various animal mice models by inhibiting mitochondrial ROS and reducing the oxidative burden in AD-like diseases [15].

Despite its neuroprotective properties, Lupeol has not received as much attention as other bioactive compounds; hence, the aim of this review is to draw attention to its role as an antioxidant and anti-inflammatory agent and its potential to treat AD. It is hoped that knowledge of the connection between OS and neurodegenerative diseases like AD with Lupeol may lead to the development of real molecular targets and new medications with the potential to be instrumental in treating diseases like AD.

## 2. Oxidative Stress and Alzheimer’s Disease (AD)

Oxidative burden is caused by a homeostatic imbalance between the ROS and antioxidant defense mechanism, which has been shown to be involved in AD pathology [16]. The important sources of cellular signaling molecules in normal homeostatic states are superoxide (O_2_^−^), hydroxyl (-OH), peroxyl (ROO), hydrogen peroxide (H_2_O_2_), and organic peroxides (R-O-O-R′), whereas abnormal accumulation may result in excessive ROS production that causes irreparable damage to the biomolecules [17]. The mitochondrion is known as the powerhouse of the cell [18] because it has the metabolic potency to synthesize adenosine triphosphate (ATP) [19] through oxidative phosphorylation, which involves the transfer of electrons from high-energy substrates to oxygen via the electron transport chain [20]. ATP is the source of energy to drive and store a variety of vital activities at the cellular level [21]. Mitochondria are stress sensitive [22], where ROS are created as a result of abnormalities in several enzymes, including α-ketoglutarate dehydrogenase complex (KGDHC) [23] and pyruvate dehydrogenase complex (PDHC) [24], which play a pivotal role in denaturing their metabolic system [25,26]. Additionally, the degradation of the promoter of the encoding gene of mitochondrial synthase enzyme (due to oxidative damage) leads to the excessive generation of free radicals, causing mitochondrial abnormalities and ATP depletion [27].

The development of AD was observed in the APP/PS1 mouse model, where the mitochondrial ROS were reported to be involved in AD pathology [28]. Similarly, other studies have reported the involvement of mitochondrial ROS in AD pathology by inducing an oxidative burden in the brain, leading to synaptic damage and cognition [29]. Furthermore, the enhanced ROS-induced oxidative damages including lipid peroxidation, protein oxidative damage, and glycoxidation was observed in AD patients [30]. Moreover, evidence from an in vitro study also confirmed ROS the leading agent of neuronal dysfunction, including AD pathology [31]. Additionally, the evidence of the progression of AD pathology due to OS was further confirmed by an in vivo study where the administration of a nanoparticle-based approach in mice exhibited the inhibition of the neuronal ROS [32]. Overall, these findings reveal that ROS triggers an imbalance in the antioxidant system which is the causal pathway for the progression of AD.

### 2.1. Contributory Sources of Oxidative Stress in AD

As OS is a major hallmark of AD like neurodegeneration [33], studies have reported other factors were also involved in boosting the ROS level, e.g., abnormal Aβ deposition, glial cell activation, homeostatic imbalance of nutrients (trace minerals zinc, iron, and copper) [34], altered cellular signaling pathways, [35] and abnormal nuclear factor erythroid 2-related factor (Nrf2) signaling [36].

#### 2.1.1. Accumulation of Abnormal Amyloid Beta

Aβ_1–42_, which is obtained as a result of the proteolytic cleavage of amyloid precursor protein (APP) by different secretases (amyloidogenic pathway), is reported to be involved in causing oxidative burden in AD [15]. The abnormal Aβ peptides causes OS, which has been shown to initiate calcium dyshomeostasis in mice brains, activating N-methyl-D-aspartate (NMDA) receptors [37]. The excessive accumulation of free radicals, due to the abnormal Aβ metabolism, damage numerous biomolecules including proteins, unsaturated fatty acids, and mitochondrial DNA. This can lead to toxicity in different cellular signaling pathways such as pathogen invading defense system, gene transcriptional regulation and other enzymatic activities, which are the basic etiological factors in AD [38].

#### 2.1.2. Activation of Glial Cells

Microglia and astrocytes are the basic safeguards for the central nervous system (CNS), as they facilitate the clearance of Aβ in the brain and maintain the permeability of the blood–brain barrier (BBB) [39,40]. Upon the over-activation of glial cells, through OS because of abnormal deposited Aβ protein or lipopolysaccharides (LPS), the inflammatory cytokines and mediators including interleukin (IL)-1β, IL-6, and interferons (IFNs), nuclear factor kappa-light-chain-enhancer of activated B cells (NF-κB), tumor necrosis factor alpha (TNF-α), and nitric oxide synthase (NOS) are released. These inflammatory cytokines and mediators initiate neuroinflammatory cascades, neuronal dysfunction, and synaptic and memory abnormalities which have been shown to be linked with progression towards AD pathology [15,41]. Similar activation of the glial cells was reported to be involved in inflammatory cascade by using APP/PS1 mice and an AD brain, where the enhanced expression of IL-1β and IL-6 was observed [42].

#### 2.1.3. Abnormal Cellular Pathways

An important consequence of the loss of nerve cells is altered neurotransmission and OS is one of the major players in abnormal neurotransmission [43], as numerous enzymatic metabolic dysfunctions and their abnormal activation were reported to be provoked by OS in different signaling pathways. For example, the stress-activated protein kinase (SAPK) pathways play a pivotal role in mediating the stress signals into the nucleus. Therefore, these pathways initiate different kinds of activated protein signaling such as ROS/protein kinase C (PKC)-dependent NF-κB and mitogen-activated protein kinase (MAPK) signaling pathways that are reported to be involved in the accumulation of abnormal Aβ peptides [44]. Additionally, other studies also highlighted the toxic role of OS that are enhancing the expression of c-Jun N-terminal kinase (JNK)/p38 levels, related to the deposition of neuritic plaques and neurofibrillary tangles in AD [45]. To prevent neural dysfunction, the activation of endogenous antioxidant mediators is crucial and, in AD, the inflammatory and oxidative hypotheses appears to play a significant role [46]. The electron transport chain’s byproducts, such as hydrogen peroxide radicals, superoxide radicals, and hydroxyl radicals, are mostly where the oxidative pathways pass through [47] and nuclear factor erythroid 2-related factor 2 (Nrf2) appears to operate as an upstream mediator to control these pathways/mediators. In turn, antioxidant response elements (ARE) and Kelch-like ECH-associated protein-1 (Keap1) tightly regulate Nrf2 [48,49]. As a result of Keap1 alteration brought on by oxidative stress, the Keap1/Nrf2 connection is broken and Nrf2 degradation is prevented. This causes Nrf2 to translocate into the nucleus, bind to ARE, and then activate antioxidant enzymes. The production of inflammatory mediators such as IL-1, IL-6, TNFs are also boosted by increasing the levels of oxidative stress, phosphatidylinositol 3-kinases (PI3K)/protein kinase B (PKB)/mammalian target of rapamycin (mTOR) [50,51]. Lastly, pro-inflammatory cytokines generated during inflammation cause synapse loss and neuronal damage that contributes to the course of AD [36].

## 3. Bioactive Compounds and Their Role in AD as Antioxidants

A healthy lifestyle through balanced diet, which contains various bioactive compounds, plays a pivotal role in reducing the risk of AD dementia [52]. Due to the lack of sufficient knowledge, the jury is out on these compounds, their availability in food, chemical forms, and their role in neuroprotection. However, some of these natural compounds and their roles as antioxidants in neuroprotection have been reported in some observational epidemiological studies and experimental research, annotating the proper molecular signaling mechanism and role in neuroprotection, especially in AD [53,54].

Antioxidants have been reported to be involved in homeostatic balance in different biological activities. These natural or synthetic antioxidants include glutathione peroxidase, Catalase (CAT), glutathione reductase, Superoxide Dismutase (SOD), Nicotinamide Adenine Dinucleotide Phosphate (NADPH), vitamin C, mannitol, bilirubin, and Glutathione (GSH), β-carotene, which play a major role in ROS reduction in AD [9]. Numerous natural antioxidants that are available in food, such as Lupeol from tomato [15], catechins, and theaflavins from tea, and curcumin from turmeric are the major focus of researchers due to their wide range of biological functions, including neuroprotection in AD [55]. For example, mitochondrial ROS generate free radicals which are counteracted by the natural antioxidant defense system. However, these natural antioxidant defense mechanisms fail in the event of excessive oxidative damage because the metabolic machinery in the cell is unresponsive. To cope with this damage, researchers are investigating different types of strategies, e.g., the use of natural bioactive compounds extracted from different medicinal plants that are used as antioxidants because of their easy accessibility, cost-effectiveness, and versatile bioactivities, such as scavenging or nearly inhibiting ROS and free radicals in different health issues, including AD [56,57].

## 4. Lupeol as an Antioxidant and Anti-Inflammatory Agent (Neuroprotective Features)

### 4.1. Antioxidant Potentials of Lupeol

Numerous naturally occurring plants, including onion, oranges, apple, and tea, have been used to extract bioactive compounds that are used as antioxidants to treat neuronal dysfunction, out of which triterpenoids are playing a pivotal role in different types of chronic disorders, including neurodegenerative diseases such as AD, Huntington’s disease (HD), and Parkinson’s disease (PD) [11,58]. These compounds are metabolic derivatives of the oligomeric isopentenyl pyrophosphate (phytochemicals). Lupeol is a pentacyclic triterpenoid and has many biological functions. It is available in fruits (e.g., mango, fig, strawberry, and red grapes), vegetables (e.g., white cabbage, pepper, cucumber, and tomato), and numerous other medicinal plants [13,14]. Lupeol has shown beneficial effects against numerous health issues, including anti-cancer, anti-microbial [59], anti-diabetic [60], cardio [61], and hepatoprotection [62] (Table 1).

Evidence has declared the beneficial antioxidant features of Lupeol in streptozotocin (STZ)-induced hyperglycemic rats’ model, where the expression of Superoxide Dismutase 2 (SOD-2) and Heme Oxygenase-1 (HO-1) were noticeably increased by the treatment of Lupeol [13]. A study was conducted where the antioxidant effect of Lupeol was investigated in the streptozotocin (STZ) and aluminum chloride (AlCl_3_)-induced male Sprague–Dawley rat model. The improved antioxidants in cortex cerebellum, such as, CAT, SOD, thiobarbituric acid reactive substances (TBARS) and GSH were found [60]. The antioxidant potentials of Lupeol and its derivatives (isolated from the stem bark of crateva nurvala) were further confirmed in triton-induced hyperlipidemic adult male rats of the Charles Forest strain, where the reduced superoxide anions and hydroxyl free radicals were found by the administration of Lupeol and chalcone (derivatives of Lupeol) [63]. Furthermore, Santiago et al. also confirmed the antioxidant nature of Lupeol (extracted from Ficus pseudo Palma Blanco) (Moraceae) against nitric oxide (NO), hydroxyl and superoxide radical scavenging potentials [64]. Similarly, Lupeol was demonstrated in diabetic rats to play a role in hepatic glucose metabolism. An enhanced and improved liver glucose level was found, including antioxidant functions [65]. Similarly, a study was carried out by Sunitha et al. to find the antioxidant capability of Lupeol and its chalcone Lupeol Lineolate by oral administration on the hepatotoxicity in the rat model. Significant improvement was observed in antioxidant level in the liver [66].The fetal cardiotoxicity induced by oxidative stress was inhibited by Lupeol, its ester, and Lupeol linoleate administration in cyclophosphamide-treated experimental rats, where the antioxidant potency was also exhibited by Lupeol [67]. Reduced oxidative abnormalities and improved enzymatic SOD, GPx and non-enzymatic GSH, ascorbic acid, vitamin E and antioxidants by the treatment of Lupeol and its linoleate ester derivative were reported in the early stage of hypercholesterolemic atherosclerosis in rats [68].

#### Role of Lupeol as an Antioxidant in Neuroprotection

**Figure 1:**
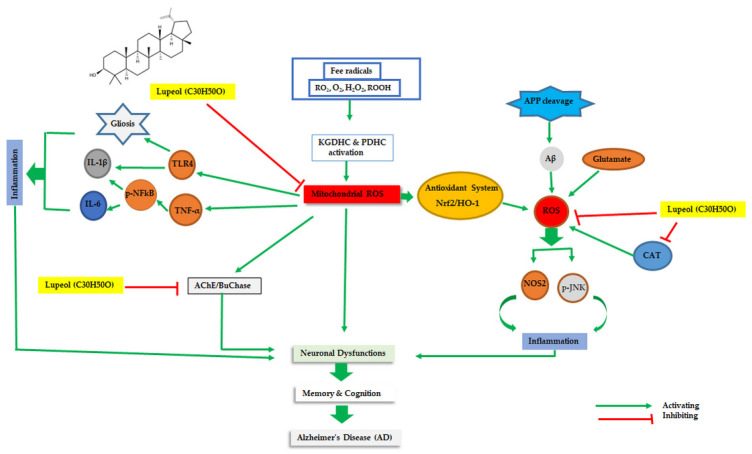
Neuroprotective effects of Lupeol against oxidative stress in AD pathogenesis. The activations of KGDHC and PDHC due to the excessive burden of free radicals are inducing oxidative stress, which is leading to the downregulation of the antioxidant defense system. Also, the overburden of AChE/BuChase and abnormal deposition of amyloid beta(Aβ) protein, due to the cleavage of amyloid precursor protein (APP), are inducing an elevated ROS level, where the inflammatory cascade is initiated due to activation of inflammatory cytokines that are proceeded by neuronal inflammation, initiating AD pathogenesis. Lupeol treatment annotated at different doses at different intervals of time was able to improve memory and cognition by inhibiting the over burden of oxidative stress and neuroinflammation.

The antioxidant and neuroprotective activities of Lupeol have been well reported in cognitive deficit, and neurochemical and biochemical abnormalities in rats as well [69] (Table 1; Figure 1). The nano-based delivery approach was used, where the antioxidant and neuroprotective potentials of Lupeol were investigated in an ischemic brain [70]. Since it is a naturally abundant triterpenoid, its medicinal outcomes were well studied [71]. For example, one study has shown a free radical scavenging activity of Lupeol since it has the potentials to donate electron/hydrogen in its structure [72]. The antioxidant potentials of Lupeol (oral administration of Lupeol at a dose of 50 mg/kg for two weeks) are well observed in Aβ-induced neuronal dysfunction in a mouse model, where Lupeol was observed to be involved in reducing oxidative stress and memory impairments by enhanced expression of Nrf2, HO-1 level [15]. Similarly, traumatically brain-injured mice were treated with Lupeol (50 mg/kg/day/mice/p.o.) to check their antioxidant potentials, where the oxidative stress and ROS level were observed to be reduced by Lupeol treatment [73]. Furthermore, Lupeol was treated against a STZ+AlCl_3_-induced diabetic and AD rat model to observe its antioxidant features and a significant improvement was found in cognition and memory impairments [60]. A study was conducted where Lupeol was investigated for its antioxidant and neuroprotective potentials against Aβ_1–42_-induced oxidative burden and neurodegeneration in mice model. The expression level of some of the markers related to oxidative stress such as Nrf-2 and HO-1 were found to be upregulated by the treatment of Lupeol (at a dose of 50 mg/kg) [15]. A similar study was carried out by Zhang et al. in middle cerebral artery occlusion (MCAO) ischemic rats, where oxidative stress and the neuroprotection of Lupeol (6 mg/mL dissolved in olive oil) was observed, while a significant improvement in activated Nrf2 and inhibited phosphorylated p38 MAPK was found [74].

**Table 1:** Antioxidant features of Lupeol.

Source	Mechanism	Model	Reference
Crataegus oxyacantha	↓NF-*κ*B, Vegf-A, IL-6, ↑FGF-2,TGF-β1, ↑collagen III, ↑IL-10	Streptozotocin-induced hyperglycemic rats	[13]
Mango Pulp, Egg, Plant, Cucumber, and Soybean	↑NO, ↓Mg^2+^,Ca^2+^ ↓endonuclease III	*Escherichia coli*	[59]
Hedera Nepalensis crude extract (HNC)	↑CAT, SOD ↓GSH, dopamine, serotonin	In vivo STZ + AlCl_3_-induced diabetic AD	[60]
Medicinal Plants	↓NRCMs, ANP, ↓BNP, ↓β-MHC, NF-κB p65	In vivo and in vitro cardiac hypertrophy in neonatal rat’s cardiomyocytes (NRCMs)	[61]
Vegetables, Edible Fruits	↓IκBα	In vivo dextran sulfate sodium (DSS)-induced acute colitis	[75]
Fruits, Vegetables	↓TGFβ1, ↑Nrf2	In vivo LPS/D-galactosamine(D-GalN)-induced liver injury	[62]
Stem Bark of C. Nurvala	↓TC, PLTAG	In vivo triton-WR 1339-induced hyperlipidemia.	[63]
Ficus pseudopalma Blanco (Moraceae)	↓NO	Ethanolic leaf extract of F. pseudopalma	[64]
Cassia Fistula	↓MDA ↑SOD, GSH, CAT	In vivo anti-diabetic study l	[76]
Banana Flower	↑SOD, CAT, GPx ↑GSH, VitC, VitE	In vivo hyperglycemic model	[65]
Medicinal Plants	↑SOD, GST, G6PD, GSH, ↑GPX, γ-GT	In vivo chronic cadmium exposure in kidney	[77]
Stem Bark of C. nurala	↑SOD, CAT, GPx, ↑G6PD, GST, GR, ϒ-GT	In vivo cadmium-induced hepatotoxicity	[66]
Mango Pulp Extract (MPE)	↓ROS, ↑Cu, Zn-SOD, CAT, GR and GST	In vivo androgen-induced oxidative stress in prostate.	[78]
Crataeva Nurvala Stem Bark	↑GSH, Vit C, Vit E	In vivo CP-induced cardiotoxicity	[67]
Crataeva Nurvala Buch-Ham (Capparidaceae)	↓TC, TG, LDL VLDL ↑HDL	In vivo hypercholesterolemic atherosclerosis	[68]
Betula Alnoides	↓AChE, MDA, nitrite, ↑GSH	In vivo amyloid beta-induced neuronal damage	[69]
Bombax Ceiba	↑T_gel_ ↓rhodamine-B, 5,6-carboxyfluorescein	Molecular modeling studies, X-ray diffraction data and FTIR studies	[71]
Crateva Adansonii Oliv. (Capparidaceae)	↓MDA, ↑CAT, GSH	In vivo CCl4-induced hepatotoxicity	[72]
Fruits, Vegetables, and Herbs	↓Aβ, ↓NOS2 ↑Nrf2, HO-1	In vivo Aβ-induced AD	[15]
Vegetables, Fruits	↑Nrf2, HO-1	In vivo traumatic brain injury	[73]
Vegetables and Fruits	↑Nrf2, ↓P38	In vivo ischemic toxicity	[74]

Abbreviations: Nuclear Factor-κB (p-NF-κB); Vascular Endothelial Growth Factor-A (Vegf-A); Fibroblast Growth Factor-2 (FGF-2); Transforming Growth Factor Beta-1 (TGF-β1); Interleukin-6 (IL-6); Nitric Oxide (NO); Magnesium (Mg^2^); Calcium (Ca^2^); Catalase (CAT); Superoxide Dismutase (SOD); Glutathione (GSH); Neonatal Rat Cardiomyocytes (NRCMs); Atrial Natriuretic Peptide (ANP); Brain Natriuretic Peptide (BNP), β-myosin Heavy Chain (β-MHC); Nuclear Factor of Kappa Light Polypeptide Gene Enhancer in B-cells Inhibitor, alpha (IκBα); Transforming Growth Factor Beta 1 (TGFβ1); Nuclear Factor Erythroid 2–related Factor 2 (Nrf2); Glutathione (GSH); Glutathione S Transferase (GST); Nitric Oxide (NO); Malondialdehyde (MDA); Total Cholesterol (TC); Glutathione Peroxidase (GPx); Glucose-6-Phosphate Dehydrogenase (G6PD); Glutathione Reductase (GR); Copper (Cu); Zinc (Zn); Low-Density Lipoprotein (LDL); Haemoxygenase (HO-1); Very-Low-Density Lipoprotein (VLDL); High-Density Lipoprotein HDL, Acetylcholinesterase (AChE); Gel-to-sol Transition Temperature (T_gel_), Amyloid Beta (Aβ); Beta-secretase 1 (BACE1); Glial Fibrillary Acidic Protein (GFAP); Ionized Calcium-Binding Adapter Molecule (1Iba-1); Cyclooxygenase-2 (COX-2); Caspase-3 (Casp-3), Bcl-2-Associated X Protein (Bax); Cytochrome C (Cyt C). The symbols ↓ and ↑ are representing the expression level of proteins to be decreased and increased respectively.

### 4.2. Anti-Inflammatory Potential of Lupeol

Chronic inflammation produces excessive cytokines and chemokines that develop a chronic pathological condition [79]. Lupeol has shown anti-inflammatory effects in numerous pathological insults [61,80], including neuronal inflammation [74] (Table 2). The anti-inflammatory potentials of Lupeol were observed in skin wound healing in rats by the modulation of NF-kB and Ki-67 [81]. Lupeol treatment was also reported to be involved in anti-inflammatory activities of allergic airways in a murine model [82]. To confirm the anti-inflammatory potentials of Lupeol, a study was performed where the activation of inflammatory markers toll-like receptor-4 (TLR4) and NF-κB were found in an osteoarthritis rat model, while Lupeol treatment attenuated these inflammatory agents and exhibited its anti-inflammatory effects [83]. The inflammation induced by LPS in retinal pigment epithelium cells (ARPE-19) was recovered by the intravitreal injection of Lupeol (100 µM) in non-infectious uveitis rats [84]. Moreover, the intraperitoneal administration of Lupeol (10, 25, or 50 mg/kg) was found to be effective against inflammatory cytokines such as tumor TNF-α, IL-1, and IL-6, in cerulein-induced acute pancreatitis in mice [85]. Lupeol (50, 100 mg/kg) was used to alleviate the inflammation brought due to the activation of TLR4 in a mouse model of viral myocarditis induced by coxsackie virus B3 (CVB3) [86]. Moreover, a study was conducted where the inhibitory and anti-inflammatory effects of Lupeol were observed in inflammatory bowel disease by inhibiting M1 and boosting M2 macrophages. The reduced expression of the pro-inflammatory cytokines, including IL-12, IL6, IL-1β and TNF-α, and an enhanced production of IL-10, an anti-inflammatory cytokine, were found by the oral administration of Lupeol (50 mg/kg, q.d.) [87]. In vivo and in silico approaches were used to show the anti-inflammatory scope of Lupeol isolated from Indian traditional plant Crateva adansonii, where its inhibitory and anti-inflammatory effects against key molecules of inflammation such as MPO, PGE_2_, and eight pro-inflammatory cytokines were found to be effective [88]. However, since in silico studies take the form of computational analysis, they need to be validated through an in vitro/vivo model. Lastly, Lupeol (10 μg/ear) was used to investigate its anti-inflammatory activities in a mouse model of skin inflammation [89]. The acute ear edema was reduced by 51 ± 7% using Lupeol (10 μg/ear).

#### Anti-Neuroinflammatory Features

The neuroprotective and anti-neuroinflammatory features of Lupeol were well studied against LPS-induced neuroinflammation via the p38/JNK pathway in mouse brain [41]. Similarly, the anti-inflammatory activity of Lupeol (extracted from constituents of Pyrus pyrifolia fruit) on the LPS-induced nitric oxide (NO) production and the expression of inducible nitric oxide synthase (iNOS) and cyclooxygenase-2 (COX-2) in macrophages and microglia were confirmed in an in vitro work, where the protein expression level of iNOS and COX-2 were increased due to NO induction, while Lupeol treatment (in a dose-dependent manner over a concentration range from 2.5 to 10 µg/mL) significantly inhibited the iNOS and COX-2 expression [90]. This finding suggests that Lupeol treatment is able to inhibit the NO level in macrophages and microglia via different mechanisms in neurodegenerative disease to reduce neuronal inflammation, including AD. The activation of stress-activated protein kinases, including p38-MAPK and JNK were induced by LPS and generate oxidative burden. Moreover, the phosphorylated p38 and JNK further activate the transcription factor activator protein (AP)-1, leading to the initiation of numerous other inflammatory genes such as IL-1, IL-2, CD40, TNF-α, and c-Jun, ultimately causing cell death. Lupeol administration brought a significant reduction in p-p38 and p-JNK protein levels [41]. Similarly, the anti-inflammatory and neuroprotective effects of Lupeol in LPS-induced neuronal inflammation in primary cerebellar cultures were also investigated, and RT-qPCR analysis exhibited the downregulation of the mRNA expression for TNF-α-, iNOS- and NLRP3-like proinflammatory markers, while a gradual reduction in the production of NO was also observed [91]. Lupeol (200–500 ng) extracted from *Celastrus paniculatus* (10 mg/mL), commonly called black oil plant, was used in the treatment of Parkinson’s disease (PD), which exhibited differential neuroprotective and protein-aggregation-mitigating effects in *C. elegans*. Later, the highest percentage of neuroprotection, including an improved nervous system performance and a reduction in the level of symptoms of PD, was observed [92]. Moreover, the anti-inflammatory and neuroprotective features of Lupeol in traumatic brain injury were also reported via the Nrf2/HO-1 pathway in a mouse model [73]. The in vitro inhibition of monoamine oxidase A and B (MAO-A and -B) in mouse macrophages exhibited the neuroprotective and anti-inflammatory potentials of Lupeol [93]. Similarly, the anti-inflammatory potentials of Lupeol (6 mg/mL) were further investigated in a middle cerebral artery-induced cerebral ischemia in rats involving Nrf2 and P38 MAPK modulation [74]. In this study, they also studied the cell viability assay to know about the toxicity features of this compound. The inflammatory effects produced due to the LPS-induced NO were investigated by the treatment of Lupeol (isolated from *Pueraria lobata* roots) in LPS-stimulated RAW 264.7 cells, where Lupeol was found to induce anti-inflammatory effects [94]. Lupeol (12.5, 25, 50, 100 mg/kg) reduced cerebral ischemia–reperfusion damage in relation to PI3K/Akt pathway modulation [95]. The better BACE1 binding affinity and BACE1 inhibitory potentials of Lupeol (obtained from *Leea indica*, and *Pueraria lobata* roots) as compared to ursolic acid [96] and quercetin [97] were observed.

In short, in accordance with the all these findings regarding the inhibitory role of Lupeol in different inflammatory signaling pathways, including the neuro- inflammation and neuroprotective mechanism in different neurodegenerations, it can be expected that a new window of observational study and research can be opened to investigate the neuroprotective and anti-inflammatory potentials of Lupeol for the inhibition of numerous neuronal diseases (Table 2).

**Table 2:** Anti-(neuro)inflammatory potentials of Lupeol.

Source	Mechanism	Model	Reference
Edible Plants, such as Olive, Fig, Mango, Carrot, Soybean	↓NF-kB, ↑Ki-67	Skin wound healing in rats	[81]
Stembark of D. Ferruginea Benth.	↓IL-4, IL-5, IL-13	In vivo animal model of allergic airway inflammation	[82]
SHM Herbs	↓TLR-4, NF-ĸB, IL-1	In vivo rats with osteoarthritis	[83]
Maytenus Salicifolia Reissek	↓IL-6, IL-8	In vitro rodent model of pan uveitis	[84]
Vegetable Oils, Cereals, Fruits	↓TNF-α, IL-1β, IL-6	In vitro cerulein-induced acute pancreatitis in mice	[85]
Vegetables, Fruits	↓TLR4, TNF-α, IL-1β	In vivo mouse model of viral myocarditis induced by coxsackie Virus B3 (CVB3)	[86]
White Cabbage, Pepper, Cucumber, Tomato, and Fruits such as Olive	↓IL-12, IL6, IL-1β, TNF-α, CD86 ↑IL-10, ↑CD206	In vivo and in vitro DSS-induced colitis mouse model	[87]
Crateva Adansonii Leaf Extracts	↓TNF-α, IL-1, IL-6	In vivo and in silico approaches in rats	[88]
Crude extract of Cariniana domestica Fruit Peels (CdE),	↓MPO activity ↓Ear edema, inflammatory cell infiltration	Mouse model of skin inflammation	[89]
Pyrus Pyrifolia	↓iNOS, COX2	LPS-activated RAW 264.7 and BV2 cell lines	[90]
Olives, Mangos and Strawberries	↓Bax, Cyt-C, caspase 9 ↓caspase 3, TNF-α, iNOS, IL-1β ↓ p-JNK, P38	In vivo LPS-induced neuronal dysfunctions	[41]
Olea Europaea (Olive), Mangifera Indica(Mango)	↓TNF, iNOS and NLRP3 ↓IL-6 mRNA	In vitro LPS-induced neurodegeneration	[91]
*Celastrus paniculatus* (CP)	↓α-syn	In vivo C. elegans PD model	[92]
Vegetables	↑Nrf2/HO-1 ↓Iba-1, GFAP ↓p-NFkB, TNFα, COX-2, IL-1 ↓Casp3, Cyt-C, BAX/BCL2	In vivo TBI-induced neurodegeneration in male wildtype C57BL/6 N mice	[73]
Vitex Grandifolia	↓ iNOS, NFkB	In vitro MAO-A and B-induced neuronal dysfunction and neuroprotection in mouse macrophages	[93]
Peppers, Tomatoes, Olive Oil	↑PI3K/Akt	In vivo cerebral ischemia–reperfusion injury in rat model	[95]
Vegetables and Fruits	↑Nrf2, ↓BAX/Bcl-2, caspase-3 ↓p38 MAPK	In vivo middle cerebral artery occlusion (MCAO) followed by reperfusion (MCAO/R)-induced cerebral ischemia.	[74]
Medicinal Plants	↓Antioxidant Activities	In vivo middle cerebral artery occlusion (MCAO)-induced ischemic stroke(nano approach)	[70]
Pueraria Lobata Roots	↓NO, iNOS, COX-2 ↓ ROS	In vitro study of RAW 264.7 murine macrophages	[94]
Peppers, Tomatoes, Olive oil, Figs	↑p-PDK1, p-Akt, pc-Raf, p BAD, ↓Casp-3	In vivo cerebral ischemia–reperfusion injury in rats	[95]

Abbreviations: Nuclear factor-κB (p-NF-κB); Interleukin-1 beta (IL-1β); Toll-like receptors (TLR-4), Tumor necrosis factor alpha (TNF-α); Cluster of Differentiation 86 (CD86); Myeloperoxidase (MPO); Inducible nitric oxide synthase (iNOS); Phosphorylated Cyclooxygenase-2 (COX-2); Bcl-2 Associated X-protein (BAX); Phosphorylated c-Jun N-terminal Kinase (*p*-JNK); NLR Family Pyrin Domain Containing 3 (NLRP3); Cytochrome C; Phosphoinositide 3-kinases (PI3K); Alpha-synuclein (α-syn); Interleukin 6 (IL-6); Nuclear factor erythroid 2-related factor 2 (Nrf2); Heme oxygenase 1 (HO-1); p38 mitogen-activated protein kinases (p38); Glial fibrillary acidic protein (GFAP); Ionized calcium-binding adapter molecule 1 (IBA1); Reactive oxygen species (ROS), Phosphoinositide-dependent protein kinase 1 (PDK1), Phosphorylated-protein kinase p-Akt, proapoptotic BH3-only protein BAD. The symbols ↓ and ↑ are representing the expression level of proteins to be decreased and increased respectively.

## 5. Neuroprotective Role of Lupeol in AD

Lupeol has shown neuroprotective effects in numerous neurological diseases, including AD (Table 3). Research has shown that Lupeol reduced the Aβ-induced oxidative burden, while increasing the level of Nrf2 and HO-1 in the cortex and hippocampus of Aβ-induced mice [15]. Glutamate is claimed to be involved in the initiation of apoptosis by stimulating abnormal redox-induced oxidative stress, and the reduction of glutathione levels and Aβ which leads to neuronal ROS generation [98]. Therefore, an in vitro study investigated glutamate and Aβ-induced OS-mediated neuronal toxicity, where Lupeol, extracted from Rhinacanthus nasutus, was effective against glutamate and Aβ-induced neurotoxicity in HT-22 mouse hippocampal cells [98]. Similarly, the neuroprotective potentials of Lupeol were investigated against Aβ-induced neurotoxicity, where behavioral and memory impairments, enhanced oxidative burdens, a reduction in antioxidants enzymatic system, and an increased expression level of proinflammatory cytokines were found in Wistar rat brains, while Lupeol (25, 50, and 100 mg/kg/day per orally) was found to be effective against all these parameters [69]. Moreover, the neuroprotective and antioxidant features of Lupeol were reported against STZ+AlCl_3_ in diabetic-induced AD in a rat model, where the reduced blood glucose level, rising level of CAT, SOD and lower GSH level were observed by the oral administration of Lupeol (10 mg/kg) [60]. Although the neurotransmitter acetylcholine (ACh) is degraded at the cholinergic synaptic position, that results in the generation of the acetyl group and choline by acetylcholinesterase (AChE) and butyrylcholinesterase (BChE). This reduced level of AChE is found to be involved in originating numerous biochemical changes, including neuronal dysfunctions in AD individuals. For this reason, the inhibitory potentials of Lupeol against AChE and BChE was investigated using the docking procedures using the Genetic Optimization for Ligand Docking suit v5.4.1 [99]. Moreover, the potential of Lupeol in AlCl_3_-induced neurotoxicity in Wistar rats was observed, where the elevated levels of pro-inflammatory cytokines (IL-1β, IL-6, and TNF-α) and degenerative changes in the hippocampal brain region in AD were inhibited by treatment with Lupeol (25 and 50 mg/kg, p.o.) [100]. Moreover, BACE1 (beta-site amyloid precursor protein (APP) cleaving enzyme 1), a member of the pepsin family of aspartyl proteases, was first identified in 1991 [99]. BACE1 is abundantly expressed in several types of neuronal cells and is concentrated in neurons, oligodendrocytes, and astrocytes in the brain [101]. Previous studies have focused on its functions as the secretase that causes the synthesis of amyloid beta, which is shown in Alzheimer’s disease [102]. The inhibitory and strong binding potentials of Lupeol (obtained from Lea indica) against BACE1 was observed through the application of molecular docking and molecular dynamics-based approaches, where the binding mechanism of Lupeol and ursolic acid with BACE1 was compared by the help of induced fit docking and classical molecular dynamics, including the steered molecular dynamics mechanism. Lupeol produced a higher binding free energy (211.87 kJ/mol) than ursolic acid (−50.23 kJ/mol), showing that Lupeol binds to the BACE1 enzyme significantly stronger than ursolic acid [96]. Furthermore, in this study, the classical and steered dynamics also revealed the favorable hydrophobic interactions between the Lupeol and the residues of the flap or catalytic dyad of BACE1. Moreover, in silico docking of mangrove legends against the AD receptor protein also confirmed the binding affinity of Lupeol with Aβ and acetylcholinesterase inhibition [103]. In this study, the binding strength, hydrogen bond length, and components of amino acid and cluster-like features were observed. However, since only the in-silico approach was used in this study, in vitro or in vivo confirmation is needed to validate the in silico model. Additionally, the inhibition of BACE1 through Lupeol was explored by comparative molecular studies, where neuroprotective inhibitory capacities of Lupeol against BACE1 were found in AD by applying the enzyme kinetics study [97]. The low inhibition constant (K) value of 1.43 μmol/L indicates the binding possibilities of Lupeol with BACE1 and the inhibitory features of Lupeol.

## 6. Pharmacokinetics of Lupeol

The Pharmacokinetic parameters of a certain compound play an important role in clinical and preclinical research to develop a drug through which the theoretical details are provided for showing the use of drugs, estimate of drugs, mechanism of action and to discover the fresh tactics to change the natural products, and plan the drug transfer system [105]. Some important characteristics of the target drug includes absorption, distribution, metabolism, and excretion (ADME) inside the body. Due to the widespread pharmacological functions of Lupeol, it is likely to be a potential therapeutic agent for different kinds of disorders [106] (Table 4). These ADME features (such as drug likeness (DL) 0.78, Caco-2 1.46, and oral bioavailability (OB) 12.12%) of Lupeol were further investigated in the traditional Chinese medicine systems pharmacology database and analysis platform (TCMSP), backing its drug resemblance [107]. Furthermore, the pharmacokinetic parameters, binding to protein, and drug relations of Lupeol were investigated in both rat and mice models [108,109]. The systemic bioavailability of Lupeol was evaluated in female mice of the CD-1 strain where Lupeol was administered orally at 200 mg/kg, using the UPLCAPCI+-MS/MS procedure. In this study, different pharmacokinetic parameters were evaluated by using the mono-compartmental model, where higher values of both the AUC (area under the curve) and Cmax (maximum plasma concentration) were noticed in solid–lipid nanoparticles (SLNs). The results revealed the elimination of Lupeol through feces with a maximum elimination time of 12 h, having a value of 163.28 ± 9.83 ng/mg. Additionally, the absorption of Lupeol by animals was also better [109]. The quantification of Lupeol in rats’ plasma was investigated through LC-MS/MS method, where the mean pharmacokinetic parameters of Lupeol were measured after the intravenous (i.v) and oral injection of 1 mg/kg and 30 mg/kg doses. The AUC0-t (h × ng/mL), Cmax (ng/mL), Kel (h− 1), Tmax (h) and T1/2 (h) were calculated after the administration of 1 mg/kg dose of Lupeol, such as 21,584.53, 12,485.69, 21,866.18, 0.14, 0.08, and 4.95, respectively, while 30 mg/kg dose of Lupeol oral administration the AUC0-t (h × ng/mL), Cmax (ng/mL), AUC0-t (h × ng/mL), Kel (h − 1), Tmax (h) and T1/2 (h) were measured as, 2190.49, 133.33, 2727.52, 0.08, 4.67, and 8.66, respectively. It was concluded from the oral administration route of Lupeol that the bioavailability of Lupeol orally is less than 1% [110]. However, steps should be taken to develop strategies through which the solubility and oral availability of Lupeol can be improved. Further, the effects of solid lipid nanoparticles (SLN) on the bioavailability of Lupeol (extracted from *Ficus religiosa* L.) was investigated, where 50 mg/kg of Lupeol was administered orally and the various pharmacokinetic parameters were observed, where it was noticed that all the investigated parameters were improved by the oral treatment of SLN-loaded Lupeol at 50 mg/kg [108]. The PEGylated liposomes loaded with Lupeol were also reported by Jun Zhang et al. to have the potential to improve the pharmacokinetic parameters of Lupeol via solving the bioavailability and hydrophilicity-like parameters [111]. In this study, the intravenous administration of Lupeol (10 mg/kg) was performed in Sprague Dawley rats, where the different pharmacokinetic parameters were measured and the AUC (area under the plasma concentration–time curve), MRT (mean residence time) and t_1/2_ values were improved.

Due to insufficient research on the metabolic mechanism of Lupeol, there is a scarcity in pharmacokinetic literature of this compound. As Lupeol has a poor water solubility (PubChem Compound Summary for CID 259846, Lupeol) remedies are needed on how to increase its solubility in water and, also, drug loading should be taken into consideration while designing the formulations for the method of administration in a traditional mode. Strategies are needed to conduct more pharmacokinetic studies on Lupeol so that its viability in clinical purposes may be assessed.

### Basic Challenges and Prospects to Boost the Pharmacokinetics and Pharmacodynamics of Lupeol

Despite the limited therapeutic uses of Lupeol because of some basic limitations, including its low solubility, bioavailability and drug delivery, which were reported in [111], numerous novel advances of targeted drug transfer and advanced techniques were also introduced regarding this compound, such as Lupeol-loaded PEGylated liposomes [111], NF-kB-PLGA nanoparticles that were loaded with Lupeol [109], Lupeol-entangled chitosan–gelatin hydrogel films [112], gold nanoparticles [113], and solid–lipid nanoparticles (SLNs) [108]. Briefly, the latest remedies are expected to develop novel methods by which all the designed parameters of Lupeol can be boosted, including solubility, cell proliferation and its effectiveness against neurodegeneration, especially AD.

## 7. Safety and Toxicity Contour of Lupeol

Lupeol has been investigated in different animal and clinical studies which reported the beneficial effects, with no toxic effects [114]. The oral administration of Lupeol in a dose of 2 mg/kg had no lethal effects over mice and rats [115]. Similarly, Lupeol (50 mg/kg/day/mice/p.o.) treatment attenuated the activation of glial cells and oxidative-stress-mediated neuronal dysfunctions in a mouse model of traumatic brain injury and did not cause any harmful effects [73]. Moreover, the neurotropic and anti-inflammatory role of Lupeol(0.1 μM) was demonstrated in LPS-induced neuronal inflammation in primary cerebellar cultures, and induced neuroprotection related to altered response of astrocytes and expression of neurotrophic and inflammatory factors, where no toxic outcomes of lupeol were found [91]. Furthermore, the antioxidant activities of Lupeol against glutamate and Aβ toxicity were also reported in an in vitro model with beneficial outcomes [98]. A clinical study performed in carcinoma patients [116] also exhibited the effective beneficial capabilities of Lupeol. Meanwhile, a pilot study was performed for the treatment of actinic keratoses using birch bark extract (containing Lupeol and other compounds), where twenty-eight patients of actinic keratoses were observed in a non-randomized pilot study for two months of clinical observation, and the birch-bark-containing Lupeol was able to clear 75% of lesions in patients [117]. Similarly, a double-blind randomized placebo-controlled phase II clinical trial was observed in children having the health issue of bedwetting (enuresis), which is upsetting and stressful for the child’s life. Lupeol (stem bark extract standardized at 1.5%) was able to give the best health outcomes, including reduced frequency of nocturia, safety, quality of life, and daytime incontinency [118]. Overall, all these observational studies have provided evidence that the administration of Lupeol has no serious health concerns. Furthermore, these studies may open a new window for Lupeol in clinical studies, especially in the neuroprotection of AD.

## 8. Conclusions, Limitations and Future Remedies

To conclude, many mechanisms such as OS, aberrant Aβ-deposition, mitochondrial ROS, and gliosis contribute to neurodegeneration. OS is one of the most promising targets of researchers in today’s time to find the inhibitory therapeutic pathways for neuronal dysfunctions, including AD. Associated with synthetic compounds are limited health outcomes and higher toxicity profiles [119] in various diseases, including neurodegenerative disorders, where their neuroprotective potentials are exhibited in animal models, but fail in clinical trials [119]. Therefore, natural compounds may be preferred over synthetic compounds. Herein, we contend that the targeting of OS with Lupeol as a natural antioxidant therapeutic agent opens a reasonable window for reducing or alleviating neuronal dysfunctions. The ADME (pharmacokinetic parameters) analysis of Lupeol also suggest its therapeutic potential. A nano-based approach is also used to enhance the potentials of Lupeol in neuroprotection [70]. According to Jose David Sachez et al., 98% of the bioactive compounds have negligible BBB permeability [120], leading to their beneficial health outcomes being limited. The BBB permeability of Lupeol (isolated from Crataegus oxyacantha) was found to be effective. The anticholinesterase functions of this compound were also investigated via the molecular docking approaches [104]. However, due to lack of interest and inaccessibility to Lupeol, its pharmacokinetics, discrete molecular structural features, binding energy level, and effective dose concentration, its metabolism in CNS-like features is still missing, resulting in limited or failed clinical trials. Therefore, through extensive research analysis, the future therapeutic window for Lupeol as a neuroprotective agent can be opened, especially in AD pathogenesis.

## Figures and Tables

**Table 3:** Effects of Lupeol on the treatment of Alzheimer’s disease.

Source	Mechanism	Model	Reference
Fruits, Vegetables, and Herbs	↓Aβ, BACE1, GFAP, Iba-1 ↓p-NFkB, TNF-α, NOS2 ↑Nrf2, HO-1	In vivo Aβ-induced AD mouse model	[15]
Pueraria Lobata Roots	↓Aβ, BACE1	Comparative molecular Docking in AD studies	[97]
Rhinacanthus Nasutus	↓Glutamate and Aβ ↓ ROS	In vitro glutamate and Aβ-induced AD Mouse hippocampal HT-22 cell lines	[98]
Betula Alnoides	↓Aβ, TNFα, IL-1β, IL-6	In vivo Aβ-induced AD Male Wistar Rats	[69]
Hedera Nepalensis	↓SOD, CAT and GSH	In vivo STZ-+ALCL_3_-induced diabetes-mediated AD	[60]
Leea Indica	↓BACE1	Molecular docking and molecular dynamic based approaches in AD model	[96]
Crataegus Oxyacantha	↓AChE, BuChase	Using in vitro experimental results and the docking score in AChE-induced AD model	[104]
Desmodium Triquetrum	↓iNOS ↓IL-1b, IL-6, TNF-α ↓AChE	In vivo aluminum chloride (AlCl3)-induced neurotoxicity in AD Wistar Rats	[100]
Mangroves	↓Aβ, AChE	Molecular docking AD model	[103]

Abbreviations: Amyloid Beta (Aβ); Beta-secretase 1 (BACE1); Glial fibrillary acidic protein (GFAP); Ionized calcium-binding adapter molecule 1 (IBA1); Phosphorylated Nuclear factor-κB (p-NF-κB); Tumor necrosis factor alpha (TNF-α); Nitric Oxide Synthase 2 (NOS2); Nuclear factor erythroid 2–related factor 2 (Nrf2); Heme oxygenase 1 (HO-1); Reactive oxygen species (ROS); Interleukin-1 beta (IL-1β); Interleukin 6 (IL-6); Superoxide dismutase (SOD); Chloramphenicol acetyltransferase (CAT); Glutathione (GSH); Acetylcholinesterase (AChE); Butyrylcholinesterase (BuChase); Inducible nitric oxide synthase (iNOS). The symbols ↓ and ↑ are representing the expression level of proteins to be decreased and increased respectively.

**Table 4:** Evaluated pharmacokinetic parameters of Lupeol in different models.

Pharmacokinetics Parameters	Analytical Method/Animal Model	Reference
AUC: 9.2-folds ↑Cmax: 3.9-folds ↑T1/2: 15.3 ± 1.3 in SLN SLN enhanced AUC and Cmax and prolonged T1/2	Solid–lipid nanoparticle (SLNs) loaded with Lupeol in Rats	[108]
Tmax: 6.444 ± 0.851 h; Cmax: 8.071 ± 2.930 μg/mL. Post-administration times: stomach, 137.25 ± 19.94 ng/mg and small intestine, 99.00 ± 12.99 ng/mg. Excretion: fecal; T1/2e: 12 h post-administration (163.28 ± 9.83 μg/mg). F: 0.645 ± 0.0581	UPLC-APCI+-MS/MS in CD-1 strain of Mice	[109]
1 mg/kg dose of Lupeol i.v. administration. AUC0-t (h × ng/mL); 21,584.53 Cmax (ng/mL); 12,485.69 Kel (h − 1); 21,866.18 Tmax (h); 0.14, 0.08 T1/2 (h); 4.95 30 mg/kg dose of Lupeol orally administration. AUC0-t (h × ng/mL); 2190.49 Cmax (ng/mL); 133.33, Kel (h − 1); 2727.52 Tmax (h); 0.08, 4.67 T1/2 (h); 8.66	LC–MS/MS Wistar Rat plasma	[110]
AUC: 3.2 times higher after IV. MRT: 2.5× and t1/2: 4.1×	Lupeol-loaded PEGylated liposomes in Rat Model	[111]

Abbreviations: AUC (area under the plasma concentration–time curve); Cmax (plasma concentration); SLN (solid liquid nanoparticle); Tmax (maximum time); Kel (apparent elimination rate constant). ↑—increase.
